# Real-World Clinical Management of Patients with Primary Biliary Cholangitis—A Retrospective Multicenter Study from Germany

**DOI:** 10.3390/jcm10051061

**Published:** 2021-03-04

**Authors:** Anne-Christin Beatrice Wilde, Charlotte Lieb, Elise Leicht, Lena Maria Greverath, Lara Marleen Steinhagen, Nina Wald de Chamorro, Jörg Petersen, Wolf Peter Hofmann, Holger Hinrichsen, Renate Heyne, Thomas Berg, Uwe Naumann, Jeannette Schwenzer, Johannes Vermehren, Andreas Geier, Frank Tacke, Tobias Müller

**Affiliations:** 1 Department of Hepatology and Gastroenterology, Charité Universitätsmedizin Berlin, 13353 Berlin, Germany; charlotte.lieb@charite.de (C.L.); elise.leicht@charite.de (E.L.); lena-maria.greverath@charite.de (L.M.G.); Lara.Steinhagen@outlook.de (L.M.S.); nina.wald-de-chamorro@charite.de (N.W.d.C.); frank.tacke@charite.de (F.T.); tobias.mueller@charite.de (T.M.); 2 IFI Institute for Interdisciplinary Medicine, Asklepios Klinik St. Georg, 20099 Hamburg, Germany; petersen@ifi-medizin.de; 3 Center of Gastroenterology Am Bayerischen Platz, 10825 Berlin, Germany; wolfpeter.hofmann@icloud.com; 4 GHZ-Center of Gastroenterology and Hepatology, 24105 Kiel, Germany; holger.hinrichsen@gastroenterologie-kiel.eu; 5 Liver Center Checkpoint, 10961 Berlin, Germany; heyne@leberzentrum-checkpoint.de; 6 Division of Hepatology, Department of Medicine II, Leipzig University, Medical Center, 04103 Leipzig, Germany; thomas.berg@medizin.uni-leipzig.de; 7 UBN/PRAXIS, 14059 Berlin, Germany; info@ubn-praxis.de; 8 Center of Gastroenterology Biesdorf, 12683 Berlin, Germany; schwenzer@bauchzentrum-biesdorf.de; 9 Department of Hepatology and Gastroenterology, University Hospital Frankfurt Am Main, 60590 Frankfurt am Main, Germany; johannes.vermehren@kgu.de; 10 Internal Medicine, University Hospital Wuerzburg, 97080 Wuerzburg, Germany; geier_a2@ukw.de

**Keywords:** primary biliary cholangitis, autoantibodies, ursodeoxycholic acid, treatment response, second line therapy

## Abstract

Background: Clinical practice guidelines for patients with primary biliary cholangitis (PBC) have been recently revised and implemented for well-established response criteria to standard first-line ursodeoxycholic acid (UDCA) therapy at 12 months after treatment initiation for the early identification of high-risk patients with inadequate treatment responses who may require treatment modification. However, there are only very limited data concerning the real-world clinical management of patients with PBC in Germany. Objective: The aim of this retrospective multicenter study was to evaluate response rates to standard first-line UDCA therapy and subsequent Second-line treatment regimens in a large cohort of well-characterized patients with PBC from 10 independent hepatological referral centers in Germany prior to the introduction of obeticholic acid as a licensed second-line treatment option. Methods: Diagnostic confirmation of PBC, standard first-line UDCA treatment regimens and response rates at 12 months according to Paris-I, Paris-II, and Barcelona criteria, the follow-up cut-off alkaline phosphatase (ALP) ≤ 1.67 × upper limit of normal (ULN) and the normalization of bilirubin (bilirubin ≤ 1 × ULN) were retrospectively examined between June 1986 and March 2017. The management and hitherto applied second-line treatment regimens in patients with an inadequate response to UDCA and subsequent response rates at 12 months were also evaluated. Results: Overall, 480 PBC patients were included in this study. The median UDCA dosage was 13.2 mg UDCA/kg bodyweight (BW)/d. Adequate UDCA treatment response rates according to Paris-I, Paris-II, and Barcelona criteria were observed in 91, 71.3, and 61.3% of patients, respectively. In 83.8% of patients, ALP ≤ 1.67 × ULN were achieved. A total of 116 patients (24.2%) showed an inadequate response to UDCA according to at least one criterion. The diverse second-line treatment regimens applied led to significantly higher response rates according to Paris-II (35 vs. 60%, *p =* 0.005), Barcelona (13 vs. 34%, *p =* 0.0005), ALP ≤ 1.67 × ULN and bilirubin ≤ 1 × ULN (52.1 vs. 75%, *p* = 0.002). The addition of bezafibrates appeared to induce the strongest beneficial effect in this cohort (Paris II: 24 vs. 74%, *p* = 0.004; Barcelona: 50 vs. 84%, *p =* 0.046; ALP < 1.67 × ULN and bilirubin ≤ 1 × ULN: 33 vs. 86%, *p =* 0.001). Conclusion: Our large retrospective multicenter study confirms high response rates following UDCA first-line standard treatment in patients with PBC and highlights the need for close monitoring and early treatment modification in high-risk patients with an insufficient response to UDCA since early treatment modification significantly increases subsequent response rates of these patients.

## 1. Introduction

Primary biliary cholangitis (PBC) is a rare chronic inflammatory biliary disease characterized by the progressive destruction of intrahepatic bile ducts, leading to cholestasis and subsequent liver damage. Although immune-mediated processes are widely considered as a major underlying cause, the exact etiology of biliary inflammation still remains to be fully elucidated but likely comprises additional factors such as environmental stimuli and epigenetic factors [1]. If untreated, PBC may lead to end-stage liver cirrhosis and eventually liver failure. Orthotopic liver transplantation is the only definitive therapy for PBC patients with end-stage liver disease. Early treatment with weight-based ursodeoxycholic acid (UDCA) has been proven to extend transplant-free survival and is therefore recommended at a dosage of 13 to 15 mg UDCA/kg bodyweight (BW)/d as a standard first-line therapy by national and international PBC clinical practice guidelines [2,3,4]. These guidelines also implemented the evaluation of specific response criteria at 12 months after the initiation of UDCA treatment for the early detection of high-risk patients with inadequate treatment responses who need treatment modification. The most commonly applied Paris-I, Paris-II, and Barcelona criteria have been widely accepted for treatment monitoring including laboratory parameters such as bilirubin and alkaline phosphatase (ALP). Recently, ALP levels ≤ 1.67 × upper limit of normal (ULN)or ALP normalization and the normalization of bilirubin values (bilirubin ≤ 1 × ULN) have been proven to yield prognostic relevance [5,6]. The risk of PBC disease progression to severe liver disease in patients with PBC may also be estimated using the UK-PBC risk score, which is based on continuous variables that have been specifically developed to identify patients at an increased risk for progression to death or liver transplantation after 5, 10, and 15 years [7,8].

In December 2016, obeticholic acid was licensed as a second-line treatment option in combination with UDCA for patients with an inadequate UDCA response or monotherapy for patients with an intolerance to UDCA and is therefore recommended by current PBC clinical practice guidelines. Bezafibrates have also been shown to increase response rates in patients with an inadequate response to first-line UDCA therapy [9] and have therefore been depicted as a potential off-label second-line treatment option in combination with UDCA in the current guidelines.

There are only very limited data concerning the real-world clinical management of patients with PBC in Germany [10]. Moreover, a recent German population-based study reported the rising prevalence of PBC and deficits in their subsequent clinical management [11]. We therefore aimed to evaluate real-world diagnostic approaches, standard first-line UDCA treatment regimens and respective response rates at 12 months applying Paris-I, Paris-II, and Barcelona criteria, ALP levels ≤ 1.67 × ULN, and bilirubin ≤ 1 × ULN in a large cohort of well-characterized patients with PBC in a retrospective multicenter study from 10 independent hepatological referral centers in Germany. The real-world management of patients with an inadequate response to standard first-line UDCA treatment including the hitherto applied individual second-line treatment regimens prior to the introduction of obeticholic acid and the subsequent response rates at 12 months were also evaluated.

## 2. Methods

### 2.1. Diagnostic Criteria

The records of patients suspected to have PBC between June 1986 and March 2017 were evaluated in a large retrospective multicenter study from 10 independent German hepatological referral centers, comprising four tertiary-care university hospitals in Berlin, Frankfurt am Main, Würzburg, and Leipzig and six hepatological centers in Berlin, Hamburg, and Kiel. The study was approved by the local Ethics Committees of the Universities of Berlin, Frankfurt, Leipzig, and Würzburg and written informed consent was obtained from all participants. The diagnosis of PBC was accepted if the patients fulfilled at least two of the following criteria (1) chronic cholestasis for more than six months; (2) the presence of anti-mitochondrial antibody (AMA) titer > 1:40 or other specific autoantibodies, including sp100 or gp210, if AMA-negative (3) the histological conformation of lymphocytic destructive cholangitis and destruction of interlobular bile ducts. Patients with concomitant features of autoimmune hepatitis (AIH) as defined by the current PBC treatment guidelines (according to liver biopsy or high simplified score according to Hennes et al. or patients who fulfilled the Paris criteria for AIH-PBC-Overlap) [3,4,12] showed lower response rates to UDCA in a previous study [13]; therefore, these patients were excluded to eliminate this bias. Furthermore, patients with concomitant features of primary sclerosing cholangitis, biliary obstruction, drug-induced cholestatic liver disease, severe non-alcoholic fatty liver disease, hemochromatosis, Wilson’s disease, alpha1-antitrypsin deficiency, alcohol abuse, chronic hepatitis B, or hepatitis C were excluded from the study by extended laboratory testing and imaging including abdominal ultrasound and magnetic resonance cholangiopancreatography. All patients underwent thorough clinical exams supplemented by laboratory tests at baseline and follow-up visits every three to four months in an outpatient setting. The clinical course of disease including the development of liver cirrhosis was evaluated.

### 2.2. Baseline Characteristics

Baseline characteristics comprised sex, age at onset of therapy, weight, serum levels of alaninaminotransferase (ALT), aspartataminotransferase (AST), alkaline phosphatase (ALP), γ-glutamyltransferase (γGT), bilirubin, albumin, prothrombin time, platelet count, immunoglobulin A (IgA), G (IgG), M (IgM), anti-mitochondrial antibodies (AMA), anti-smooth muscle antibodies (SMA), anti-nuclear antibodies (ANA), anti-sp100, anti-gp210, and, if available, a histological evaluation of PBC.

### 2.3. Evaluation of Standard First-Line UDCA Treatment Regimens and Response Rates at 12 Months

Standard first-line UDCA treatment regimens in mg UDCA/kg bodyweight per day (mg UDCA/kg BW/d) were evaluated. At 12 months, the ALP, AST, and bilirubin levels were assessed to evaluate Paris-I, Paris-II, and Barcelona criteria, ALP levels ≤ 1.67 × ULN, and bilirubin ≤ 1 × ULN to define an adequate respectively inadequate UDCA response.

### 2.4. Management of Patients with an Inadequate Response to First-Line UDCA Treatment

The management of patients with an inadequate response to first-line UDCA treatment including the evaluation of hitherto applied second-line treatment regimens was evaluated. Subsequent response rates at 12 months after treatment modification were assessed by applying Paris-I, Paris-II, and Barcelona criteria, ALP cut-off levels ≤ 1.67 × ULN, and bilirubin ≤ 1 × ULN.

### 2.5. Statistical Analysis

Analyses were performed by IBM SPSS Statistic Version 24 for Windows (IBM, Armonk, NY, USA) and Prism 6.0 (GraphPad Software, La Jolla, CA, USA). Comparison between groups were made by using the Kruskal–Wallis test, Mann–Whitney test, and Fischer’s exact test. Data are presented as the median and interquartile range (IQR). The Kaplan–Meier survival curve with the Mantel–Cox test was examined to assess the relationship between the response to therapy according to Paris-II and the development of liver cirrhosis and the log-rank test for statistical assessment. This study was performed in accordance with the ethical guidelines of the 1975 Declaration of Helsinki and was approved by the local Ethics Committee (EA2/035/07; 03-2015).

## 3. Results

### 3.1. Study Population

**Figure 1:**
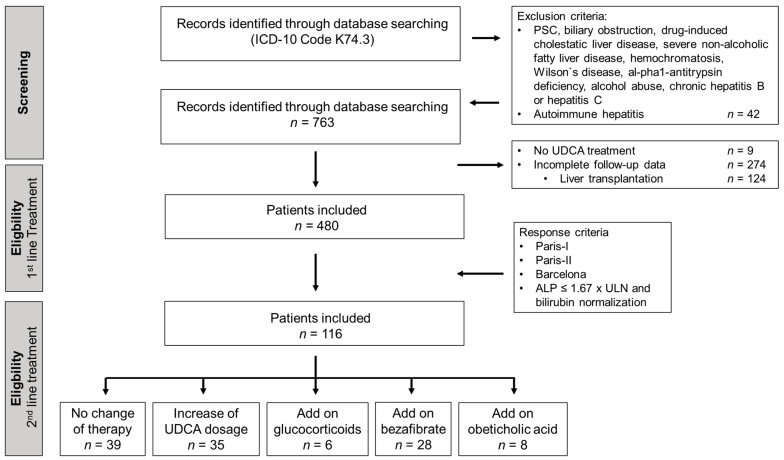
Study flow diagram. In total, 763 patients with confirmed PBC from 10 independent hepatological referral centers were screened for study eligibility and real-world first-line UDCA treatment regimens and subsequent response rates at 12 months after the initiation of treatment were evaluated in 480 patients. Patients with an inadequate response to at least one of the response criteria (*n* = 116) were evaluated for hitherto available second-line treatment regimens and subsequent response rates at 12 months after treatment modification.

As depicted in Figure 1, a total of 763 records from patients suspected with PBC was identified through an extended database search. Among them, 283 patients were excluded from this study, mainly due to subsequent incomplete data sets (150 patients), liver transplantation (124 patients) or lack of subsequent UDCA treatment (nine patients). Patients with apparent autoimmune hepatitis overlap were also excluded. Therefore, 480 patients with PBC were enrolled for first-line therapy analysis. A total of 116 patients showed an inadequate response to UDCA according to at least one criterion and were therefore included for second-line therapy analysis.

### 3.2. Baseline Characteristics

The overall study population of 480 patients with PBC comprised 431 females (89.8%) and 49 (10.2%) males (Table S1). The median age at UDCA treatment initiation was 57 years (Q1–Q3: 48 to 64 years). In 83.5% (401/480) of the patients, the diagnosis of PBC was established based on cholestatic biochemical patterns and PBC-specific autoantibodies. In total, 399 (86.9%) patients out of 459 were AMA-positive. Autoantibodies against sp100 were determined in 112 patients, of which 28 (25%) had a positive result. A liver biopsy was carried out in 26.5% (127/480) and varied largely between the different centers (17.1 to 50%).

### 3.3. Standard First-Line UDCA Treatment Regimens and Response Rates at 12 Months

In the overall cohort, the median UDCA dosage was 13.2 mg UDCA/kg BW/d and ranged between a minimum of 5 mg UDCA/kg BW/d and a maximum of 28.3 mg UDCA/kg BW/d (IQR: 3.9 (Q1–Q3: 11.1 to 15 mg UDCA/kg BW/d)).

As depicted in Table 1, at 12 months after the initiation of UDCA treatment, Paris-I criteria for adequate treatment response were met in 91% (253/278) of patients. Applying Paris-II, and Barcelona criteria, an adequate UDCA treatment response was achieved in 71.3% (201/282) and 61.3% (273/439) of patients, respectively. In total, 83% (365/440) of the patients showed ALP levels ≤ 1.67 × ULN, 95.5% (383/401) and achieved the normalization of bilirubin, and 81% (325/402) showed ALP ≤ 1.67 × ULN and bilirubin normalization. In 64 patients, full clinical follow-up data were available, allowing for the assessment of the UK-PBC risk score. In these patients, an inadequate response to UDCA at 12 months after treatment initiation according to Paris-I and Paris-II criteria was associated with a significantly higher UK-PBC risk score compared to patients with an adequate UDCA response (*p* < 0.001; Table S2). The risk of experiencing an event (increase in bilirubin value above 100 µmol/L, liver transplantation, or death) within 15 years varied from 9.80 (±8.17; Paris-II) and 18.28% (±10.98; Paris-I) among non-responders. However, when using the Barcelona criteria, patients who did not adequately respond to UDCA were not at a significantly higher risk after 15 years as compared to patients with an adequate response (8.06 vs. 7.06%; *p* = 0.423). Moreover, we evaluated the proportion of patients who developed liver cirrhosis in relation to the one-year response rate under UDCA. Overall, significantly more patients with an inadequate one-year response to UDCA according to Paris-I and Paris-II developed liver cirrhosis (Figure S1).

### 3.4. Management of Patients with Inadequate UDCA Treatment Response

As depicted in Figure 1, a total of 116 patients showed an inadequate response to the standard first-line UDCA treatment at 12 months after treatment initiation according to at least one criterion. Within this group of patients, 34% (39/116) of patients did not undergo any change of treatment and 66% (77/116) underwent treatment modification: 30% (35/116) obtained an increased UDCA dosage, 24% (28/116) obtained fibrates as an add-on therapy to UDCA, 5% (6/116) obtained glucocorticoids as an add-on therapy to UDCA and 7% (4/63) obtained obeticholic acid as an add-on therapy to UDCA.

At 12 months after the initiation of second-line therapy, Paris-I and Paris-II criteria were available in 58 patients, Barcelona criteria in 90 patients and ALP levels ≤ 1.67 × ULN and bilirubin normalization in 83 patients (Table 2). Overall, the diverse second-line treatment regimens applied led to significantly higher response rates according to Paris-II (35% vs. 60%, *p =* 0.005), Barcelona (13% vs. 34%, *p =* 0.0005), ALP ≤ 1.67× ULN and bilirubin normalization (52.1 vs. 75%, *p =* 0.002).

**Figure 2:**
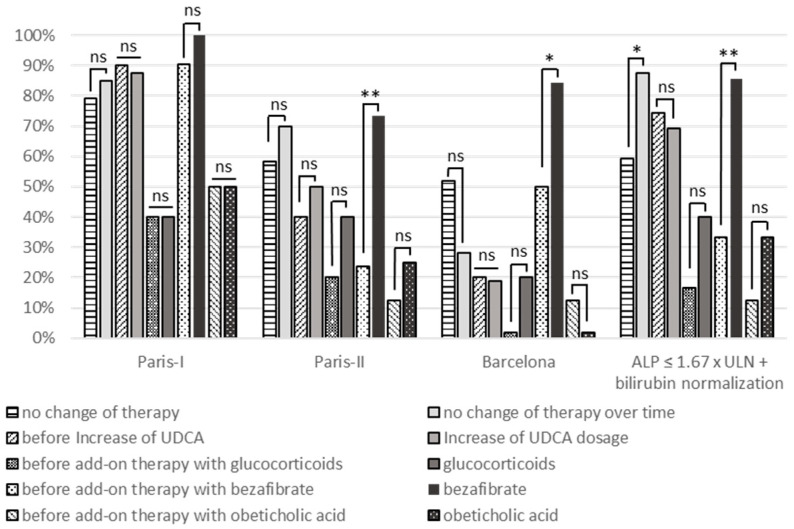
Management of patients with inadequate response to standard UDCA first-line treatment. The real-world management of 116 patients with inadequate response to standard first-line UDCA treatment according to at least one criterion is depicted, including patients without treatment modification (*n =* 39), increase of UDCA dosage (*n =* 35), addition of glucocorticoids (*n =* 6), bezafibrates (*n =* 28) or obeticholic acid (*n =* 4). Treatment response rates according to Paris-I, Paris-II, and Barcelona criteria and ALP levels ≤ 1.67 × ULN and bilirubin ≤ 1 × ULN at 12 months after treatment modification, respectively; continuation of UDCA-monotherapy was analyzed. * *p* < 0.05, ** *p* < 0.01, not significant (ns) = *p* ≥ 0.05.

As depicted in Figure 2 (Table S3), the UDCA dosage intensification and the addition of glucocorticoids did not increase response rates in patients with an inadequate UDCA response, whereas the addition of fibrates significantly enhanced response rates according to Paris-II (*p =* 0.004) and Barcelona criteria (*p =* 0.046) and ALP levels ≤ 1.67 × ULN and bilirubin normalization (*p =* 0.001) at 12 months after the initiation of treatment modification. In the small group of patients obtaining additional obeticholic acid since approval in December 2016, there was a trend towards higher response rates with respect to ALP levels ≤ 1.67 × ULN and bilirubin normalization.

### 3.5. Evaluation of Liver Biochemistry

**Figure 3:**
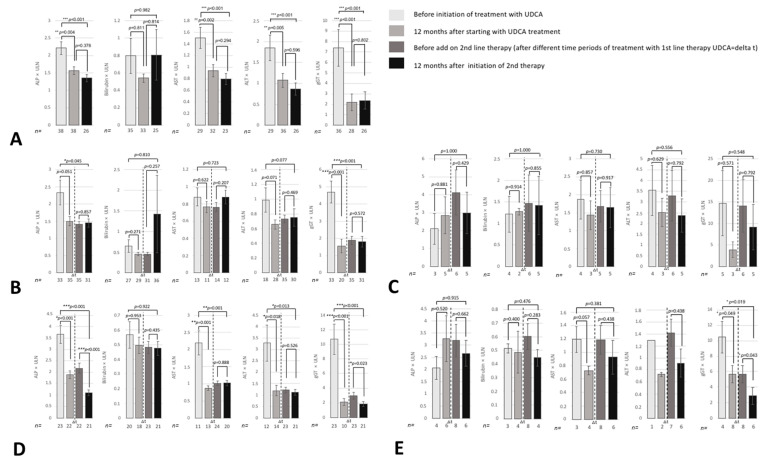
Evaluation of liver biochemistry. The course of liver biochemistry in patients with inadequate response to standard first-line UDCA treatment according to the subsequent individual management (**A**): no treatment modification (*n* = 39), (**B**): increase of UDCA dosage (*n* = 35), (**C**): addition of glucocorticoids (*n* = 6), (**D**): bezafibrates (*n* = 28) or (**E**): obeticholic acid (*n* = 4) is shown. The first bar represents the baseline values for alkaline phosphatase [ALP], bilirubin, aspartate aminotransferase [AST], alanine aminotransferase [ALT] and gamma-glutamyl transferase [gGT]. The second bar represents the laboratory results at 12 months after the initiation of standard first-line UDCA treatment. The third bar represents baseline values before the start of second-line treatment after different time periods under UDCA treatment. The fourth bar depicts the effect on liver biochemistry at 12 months after treatment modification. * *p* < 0.05, ** *p* < 0.01, *** *p* < 0.001, ns = *p* ≥ 0.05.

As depicted in Figure 3, intensified UDCA treatment and/or the addition of glucocorticoids did not improve liver biochemistry, whereas the addition of fibrates significantly reduced ALP (*p* < 0.001) and gamma-glutamyl transferase (gGT) levels (*p =* 0.023). Additional treatment with obeticholic acid also showed a reduction of gGT levels (*p =* 0.043). Bilirubin levels, which normalized in the vast majority of patients during first-line therapy, showed no further decrease irrespective of the second-line therapy applied.

## 4. Discussion

Primary biliary cholangitis may be associated with considerable morbidity despite recent advances in the management of this disease [2]. Thus, there is an unmet clinical need to improve the management of PBC, in particular in patients with an inadequate response to standard first-line treatment with weight-based UDCA. In this retrospective multicenter study, so-far the largest, we provide insight into the real-world clinical management of patients with PBC with respect to the recently revised national and international clinical practice guidelines. In this study from 10 independent hepatological centers in Germany, the diagnosis of PBC was established by serological parameters in 83.5% of all patients, i.e., chronic cholestasis and specific autoantibodies, whereas in 26.5% of all patients, an additional histological examination were carried out. Detailed analysis of the treatment regimens applied by the different centers revealed a median UDCA dosage of 13.2 mg/kg BW/d as the standard first-line therapy in the study cohort, achieving adequate treatment response rates in 60 to 90% of patients at 12 months after the initiation of therapy depending on the response criteria applied. Therefore, up to 40% of patients showed an inadequate UDCA response at 12 months after the standard UDCA first-line therapy. Of note, in almost one third of these patients in this high-risk group, there was no obvious treatment modification, whereas in the majority of cases the UDCA dosage was increased. Other treatment modifications included the off-label addition of fibrates or, less frequently, the addition of budesonide. Since 2017, the patients were also treated with obeticholic acid as the only licensed second-line therapy. Therefore, the number of patients receiving obeticholic acid was very limited. Taken together, the diverse second-line treatment regimens applied led to significantly higher response rates according to Paris-II and Barcelona criteria, and ALP ≤ 1.67 × ULN and bilirubin normalization. The addition of fibrates appeared to induce the strongest beneficial effect in this cohort. 

Several limitations of the present study need to be acknowledged. Due to the retrospective character of this study, a systematic evaluation of all biochemical parameters in every study participant was not available, leading to inconsistent patient numbers depending on the different response criteria in the overall cohort. Additionally, patients who underwent liver transplantation were excluded due to incomplete data. Another limitation of the study is that the data available on further follow-up in patients without treatment modification are limited and therefore do not allow to draw a solid conclusion. Moreover, a certain selection bias cannot be excluded since all study participants were recruited from four tertiary care centers and six hepatological referral centers, leading to a potential selection of difficult-to-diagnose cases, which likely explains the rather low proportion of patients with PBC-specific autoantibodies in the present cohort. However, a recent population-based study with non-selected PBC patients revealed no obvious difference to earlier hospital-based studies [14].

However, we would like to strengthen the fact that this is, so far, the largest retrospective multicenter study addressing the real-world management of patients with PBC in Germany, comprising patients from 10 different hepatological referral centers. The baseline characteristics of the present PBC cohort were consistent with previous PBC study populations, showing a clear predominance of female patients with a female-to-male ratio of 9:1. An increasing proportion of male patients, as postulated in a recent epidemiological study [15] and a recent German retrospective study [11], was not observed in the present cohort. The median age at treatment initiation was 57 years, which was in line with the age peak described in the literature between the fifth and seventh decade of life. Of note, depending on the individual participating center, between 5 and 16% of all PBC patients were AMA-negative, whereas previous studies described only 5 to 10% AMA-negative patients [16,17,18]. However, in a recent Polish study [19], the proportion of AMA-negative patients was 18.9%, suggesting that the proportion of AMA-negative patients in special populations might be higher than previously reported. In our first-line study, 399 of 459 patients were AMA-positive whereas only 112 cases were tested for the presence of specific autoantibodies against protein sp100 with a positive result in 25% of patients, which might be due to the fact that these antibodies are not readily available in daily routine use. However, our observation was in line with the very heterogeneous results of previous meta-analyses from 2014, describing antibodies against sp100 in 7 to 60% of all patients [20].

With respect to the applied first-line UDCA therapy, the median dosage of 13.2 mg UDCA/kg BW/d was close to the lower limit of the recommended dosage of 13 to 15 mg UDCA/kg BW/d. Adequate UDCA response rates in 91% of patients at 12 months after treatment initiation according to Paris-I criteria was higher compared to previous studies of other countries. In a Dutch study from 2009, the overall response rate according to Paris-I criteria was 66% [21]; in a French study from 2011, it was 76% [22]; and in a large multicenter British study from 2013, it was 79% [23]. This discrepancy might be explained by the large proportion of patients with early PBC stages in the present cohort defined by normal bilirubin levels prior to the start of UDCA treatment. Interestingly, a recent German prospective study from 2019 showed a similarly high response rate according to Paris-I criteria [10]. This study also described a high number of patients in an early stage of PBC. Therefore, Paris-II criteria might be more adequate to examine the response rate in the present cohort. In a recently published study, Murillo-Perez et al. highlighted that not only a reduction of ALP level, but both bilirubin normalization and an ALP level below 1 × ULN significantly contributes to a more accurate risk stratification and improved survival [6]. Therefore, not only one criterion but several criteria (the Barcelona criterion together with Paris-II criterion and ALP level ≤ 1.67 × ULN with bilirubin normalization) should be used for each patient; furthermore, a complete normalization of ALP and bilirubin level should possibly be targeted in the future to determine the lowest risk for a disease progression and, if needed, to intensify the therapy. 

In line with this hypothesis, applying Paris-II and Barcelona criteria, adequate response rates of 71 (Paris-II) and 61% (Barcelona) at 12 months after treatment initiation were consistent with previous observations [22,24].

Our findings demonstrate higher treatment response rates at 12 months after treatment modification under real-world conditions. Of note, comparison of different treatment regimens in patients with an inadequate therapy response showed particularly higher response rates among patients receiving additional fibrates. These results are in line with previous studies showing higher response rates for fibrates as an add-on therapy to UDCA [9,25,26]. In the other large cohort of the study—patients without any change of treatment regime—there was no significant improvement in treatment response according to Paris-I, Paris-II, and Barcelona criteria. However, there was a significant increase in patients achieving ALP levels ≤ 1.67 × ULN and bilirubin normalization. These results are in line with a recent study from Germany by Sebode et al., who also showed that the guideline recommended treatment regime of patients with PBC was partly not applied [11]. Follow-up data for treatment modification with obeticholic acid were missing due to the short period of this recently licensed therapy. However, we already observed a clear trend towards a better therapy response rate regarding the ALP-level ≤ 1.67 × ULN and bilirubin normalization and a significant reduction of gGT after the addition of obeticholic acid to UDCA. However, our data are in line with the findings by Nevens et al. who showed an improved response rate according to ALP levels below 1.67 × ULN and bilirubin normalization under obeticholic acid [27].

In conclusion, this large real-world multicenter study confirms high response rates following UDCA first-line standard treatment in patients with PBC and highlights the need for close monitoring and early treatment modification in high-risk patients with an insufficient response to UDCA since early treatment modification significantly increases the subsequent response rates of these patients. A large prospective observation study has already been initiated and will provide further long-term outcomes.

## Figures and Tables

**Table 1:** First line UDCA treatment response at 12 months.

Response Criteria at 12 Months of Standard UDCA Therapy	Total
**Paris-I** ALP < 3 × ULN + AST < 2 × ULN + bilirubin normalization	91.0% (253/278)
**Paris-II** ALP < 1.5 × ULN + AST < 1.5 × ULN + bilirubin normalization	71.3% (201/282)
**Barcelona** ALP ≤ 1 × ULN or reduction of ALP > 40%	61.3% (273/439)
**ALP ≤ 1.67 × ULN**	83.0% (365/440)
**Bilirubin ≤ 1 × ULN**	95.5% (383/401)
**AP ≤ 1.67 × ULN + bilirubin normalization**	80.8% (325/402)

UDCA: ursodeoxycholic acid; ALP: alkaline phosphatase; ULN: upper limit of normal; AST: aspartataminotransferase.

**Table 2:** Second line treatment response in patients with inadequate UDCA response.

Response Criteria at 12 Months of UDCA Therapy	Before Initiation of 2nd Line Therapy and After at Least 12 of Therapy with UDCA	12 Months After Initiation of 2nd Line Therapy	*p*
**Paris-I** ALP < 3 × ULN + AST < 2 × ULN + Bilirubin ≤ 1mg/dL	76.8% (53/69)	84.5% (49/58)	0.371
**Paris-II** ALP < 1.5 × ULN + AST < 1.5 × ULN + Bilirubin ≤ 1mg/dL	34.9% (24/69)	60.3% (35/58)	**0.005**
**Barcelona** ALP ≤ 1 × ULN or reduction of ALP > 40%	12.9% (13/101)	34.4% (31/90)	**0.0005**
**ALP ≤ 1.67 × ULN + Bilirubin ≤ 1 × ULN**	52.1% (50/96)	74.7% (62/83)	**0.002**

Bold values indicates a statistical significance (*p* < 0.05).

## Data Availability

Informed consent was obtained from all subjects involved in the study.

## Supplementary Materials

The following are available online at https://www.mdpi.com/2077-0383/10/5/1061/s1. Figure S1: Development of liver cirrhosis in relation to 12 months UDCA treatment response, Table S1: Baseline characteristics, Table S2: UK-PBC-Risk-Score in relation to 12 months UDCA treatment response, Table S3: Treatment response before and after 12 months of second line therapy in detail.
