# Supplementary material for: Real-World Clinical Management of Patients with Primary Biliary Cholangitis—A Retrospective Multicenter Study from Germany
Source: J Clin Med. 2021 Mar 4;10(5):1061. doi: 10.3390/jcm10051061 (PMC7961881; doi:10.3390/jcm10051061)
Supplement: Supplementary file 1 [file jcm-10-01061-s001.pdf]

Table S1. Baseline characteristics.

		Population
**Age (median, Q1-Q3)**		57 (48–64)
**Sex**	**male**	10.2% (49/480)
	**female**	89.8% (431/480)
**PBC Diagnosis**	**serological**	73.5% (353/480)
	**serological + histological**	26.5% (127/480)
**Autoantibodies**	**AMA**	86.9% (399/459)
	**ANA**	43.1% (160/371)
	**sp100**[1]	25% (28/112)
	**gp210**[2]	20% (2/10)

anti-sp100 antibody (sp100 is a nuclear antigen); gp210: Anti-glycoprotein-210 antibody.

Table S2. UK-PBC-Risk-Score in relation to 12 months UDCA treatment response.

UK-PBC Risk Score		*n*	5 years (%)	10 years (%)	15 years (%)	*p*
**Total**		64	1.26 ± 1.23	4.09 ± 3.84	7.35 ± 6.59	
**Paris I**	**Nonresponder**	7	3.29 ± 2.17	10.46 ± 6.60	18.28 ± 10.98	***0.015***
	**Responder**	57	1.01 ± 0.78	3.30 ± 2.48	6.01 ± 4.39	
**Paris II**	**Nonresponder**	20	1.70 ± 1.57	5.49 ± 4.84	9.80 ± 8.17	***0.038***
	**Responder**	44	1.05 ± 0.99	3.45 ± 3.14	6.24 ± 5.49	
**Barcelona**	**Nonresponder**	19	1.37 ± 1.14	4.47 ± 3.64	8.06 ± 6.38	*0.423*
	**Responder**	45	1.21 ± 1.27	3.93 ± 3.94	7.06 ± 6.73	

Table S3. Treatment response before and after 12 months of second line therapy in detail.

Response Criteria at 12 Months of UDCA[1]	Before Increase of UDCA	12 Months after UDCA Increase	*p*	Before Add-on Therapy with Bezafibrate	12 Months after Add-On Therapy with Bezafibrate	*p*	Before Add-On Therapy with Glucocorticoids	12 Months After add-On therapy with Glucocorticoids	*p*	Before Add-On Therapy with Obeticholic Acid	12 Months After Add-On Therapy with Obeticholic Acid	*p*	No Change in Therapy Management	12 Months After Remaining in Therapy	*p*
**Paris-I** ALP[2] < 3 x ULN[3] + AST[4] <2 x ULN + Bilirubin ≤ 1mg/dL	90% (9/10)	87.5% (7/8)	*1.000*	90.5% (19/21)	100% (19/19)	*0.489*	40% (2/5)	40% (2/5)	*1.000*	50% (4/8)	50% (2/4)	*1.000*	79.2% (19/24)	85% (17/20)	*0.710*
**Paris-II** ALP < 1.5 x ULN + AST < 1.5 x ULN +Bilirubin ≤ 1mg/dL	40% (4/10)	50% (4/8)	*1.000*	23.8% (5/21)	73.7% (14/19)	***0.004***	20% (1/5)	40% (2/5)	*1.000*	12.5% (1/8)	25% (1/4)	*1.000*	58.3% (14/24)	70% (14/20)	*0.534*
**Barcelona** ALP ≤ 1 x ULN or reduction of ALP > 40%	20% (7/35)	19% (6/31)	*1.000*	50% (11/22)	84,2% (16/19)	***0.046***	0% (0/6)	20% (1/5)	*0.455*	12.5% (1/8)	0% (0/7)	*1.000*	51.8% (14/27)	28% (7/25)	*0,097*
**ALP ≤ 1.67 x ULN + Bilirubin ≤ 1 x ULN**	74.2% (23/31)	69.2% (18/26)	*0.771*	33.3% (7/21)	85,7% (18/21)	***0.001***	16.7% (1/6)	40% (2/5)	*0.546*	12.5% (1/8)	33,3% (2/6)	*0.539*	59.3% (16/27)	87,5% (21/24)	***0.031***
**ALP ≤ 1.67 x ULN**	74,3% (26/35)	74.1% (23/31)	*1.000*	31,8% (7/22)	81%% (17/21)	***0.002***	16,7% (1/6)	40% (2/5)	*0.546*	12,5% (1/8)	16,7% (1/6)	*1.000*	71,1% (19/28)	92,3% (24/26)	***0.041***
**Bilirubin ≤ 1 x ULN**	96.8% (30/31)	92.3% (24/26)	*0.587*	100% (23/23)	95,5% (21/22)	*0.489*	50% (3/6)	60% (3/5)	*1.000*	87.5% (7/8)	80% (4/5)	*1.000*	92.6% (25/27)	77,8% (21/27)	*0.250*

1: ursodeoxycholic acid (UDCA); 2: alkaline phosphatase (ALP); 3: upper limit of normal (ULN); 4: aspartataminotransferase (AST).

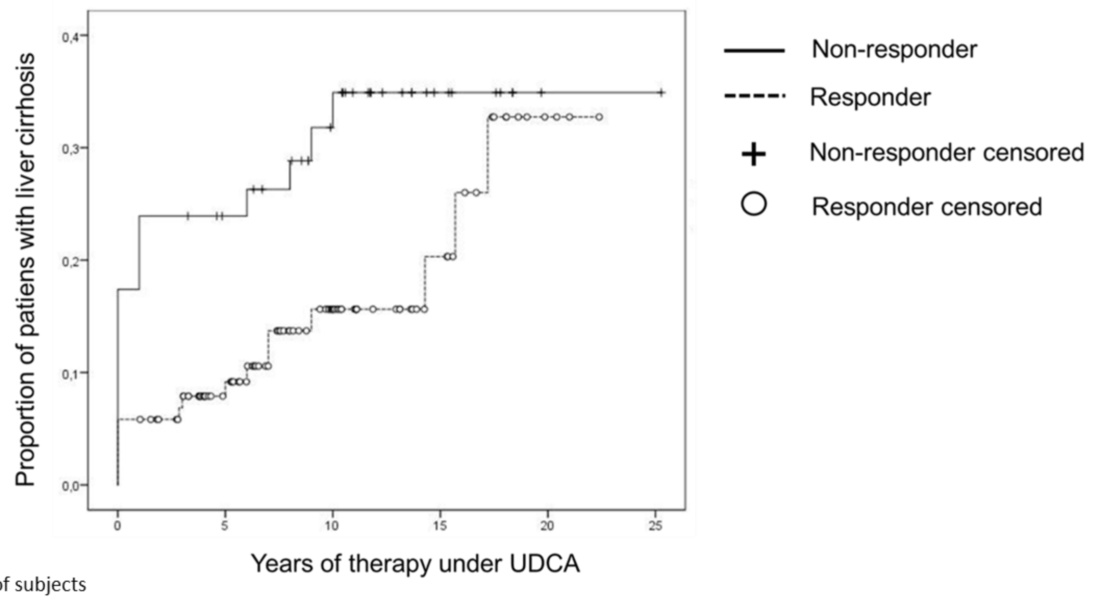

Number of subjects						
Non-responder	46	41	36	31	26	21
Responder	103	97	92	87	82	77

Figure S1. Development of liver cirrhosis in relation to 12 months UDCA treatment response. Kaplan-Meier analysis illustrating the relationship between 1-year response to therapy according to Paris-II criteria and the time of diagnosing liver cirrhosis after initiation of therapy. The proportion of patients who developed liver cirrhosis over time was increased in the group of patients with an inadequate 1-year response to UDCA (log rank: $p = 0.043$; HR: 2.3 (1.12, 4.81)).
